# 3D extruded composite thermoelectric threads for flexible energy harvesting

**DOI:** 10.1038/s41467-019-13461-2

**Published:** 2019-12-06

**Authors:** J. Peng, I. Witting, N. Geisendorfer, M. Wang, M. Chang, A. Jakus, C. Kenel, X. Yan, R. Shah, G. J. Snyder, M. Grayson

**Affiliations:** 1 Department of Electrical and Computer Engineering, Northwestern University, Evanston, IL 60208 USA; 2 Department of Materials Science and Engineering, Northwestern University, Evanston, IL 60208 USA; 3 Dimension Inx, LLC, Chicago, IL 60616 USA; 4 Department of Bioengineering, University of Illinois at Chicago, Chicago, IL 60607 USA; 5 Department of Biomedical Engineering, Northwestern University, Evanston, IL 60208 USA

**Keywords:** Electrical and electronic engineering, Thermoelectrics

## Abstract

Whereas the rigid nature of standard thermoelectrics limits their use, flexible thermoelectric platforms can find much broader applications, for example, in low-power, wearable energy harvesting for internet-of-things applications. Here we realize continuous, flexible thermoelectric threads via a rapid extrusion of 3D-printable composite inks (Bi_2_Te_3_ *n-* or *p-*type micrograins within a non-conducting polymer as a binder) followed by compression through a roller-pair, and we demonstrate their applications in flexible, low-power energy harvesting. The thermoelectric power factors of these threads are enhanced up to 7 orders-of-magnitude after lateral compression, principally due to improved conductivity resulting from reduced void volume fraction and partial alignment of thermoelectric micrograins. This dependence is quantified using a conductivity/Seebeck vise for pressure-controlled studies. The resulting grain-to-grain conductivity is well explained with a modified percolation theory to model a pressure-dependent conductivity. Flexible thermoelectric modules are demonstrated to utilize thermal gradients either parallel or transverse to the thread direction.

## Abstract (web summary)

Flexible thermoelectric composite threads are reported for wearable thermal energy harvesting platforms where rigid materials lack compatibility. Thermoelectric thread modules are demonstrated, and pressure-dependence shows thread compression to be essential for improving electrical conductivity.

## Introduction

Thermal energy harvesting from thermoelectric materials is attractive for wearable, portable, and internet-of-things electronics, even at power levels as low as microwatts^1–4^. However, conventional thermoelectric semiconductors are rigid and do not easily conform to wearable platforms^5^, so flexible thermoelectric power sources are highly desired as a critical step towards robust, wearable electronic devices. Thin-film thermoelectrics represent one strategy to achieve flexibility, and various chemical methods such as intercalation and polymerization^6,7^ or physical deposition techniques^8,9^ have been developed. Nevertheless, thin films suffer from their small, micron-scale layer thicknesses that limit the active cross-section^10^ and therefore the performance, and frequently require a substrate for mechanical strength^11,12^. Alternatively, single fibers^13^ and twisted yarns^14^ have been realized. But deposition again requires electron beam sputtering in vacuum, which is impractical at industry levels due to the high cost and low throughput. Alternately, conducting polymers composed of flexible long molecular chains have demonstrated promising thermoelectric performance^15,16^. But in practice the necessary inclusion of dopants renders them brittle^17,18^ such that they only been characterized as thin films on substrates^11^, and no *p-n* junction devices of free-standing films have been demonstrated due to their fragility.

In this context, a free-standing composite thermoelectric, on the other hand, is particularly promising, and offers a viable solution for scalable mass production with the promise of achieving figures-of-merit on a par with bulk materials. A thermoelectric composite is principally composed of functional thermoelectric filler surrounded by an inert matrix, such as a polymer adhesive that provides elasticity while serving as a binder to hinge rigid thermoelectric micrograins^19^. Although conducting polymers could provide enhanced conductivity^20–23^, a nonconductive polymer matrix allows the electrical and thermoelectric transport properties to be dominated by the TE filler, thereby achieving maximal thermoelectric performance. Bulk bismuth telluride and its alloys with antimony telluride and bismuth selenide are good candidate fillers due to their exceptional TE performance near room temperature^24,25^. Prior composite preparations such as 3D printing^26,27^ and screening printing^28^ demonstrate scalable production of such thermoelectric composites, but to date, all of these printed bulk thermoelectrics have been rigid, not flexible.

In this work, bismuth telluride micrograins are embedded within a flexible nonconductive polymer to produce continuous thermoelectric composite threads. These threads were rapidly 3D-extruded at a rate of *r* = 4 cm/s in ambient air and collected on a spool (see Supplementary Movie 1). The composite conductivity was dramatically improved after lateral compression of the threads generated by an in situ vice with controlled pressure. Percolation theory accurately describes the pressure-dependent conductivity, which exhibits a slightly super-linear power-law dependence on pressure. A flexible thermoelectric module with alternating *n*- and *p*-type legs was fabricated to demonstrate thermal energy harvesting using a scalable structure with an eye on eventual mass production.

## Results

### Beyond rigid thermoelectrics

The first step in fabricating TE composites for energy harvesting is the selection of the thermoelectric filler material. High energy conversion efficiency results from a large Seebeck coefficient *S* to increase the potential difference caused by a temperature gradient, a low thermal conductivity *κ* to minimize passive heat flow, and high electrical conductivity *σ* to minimize Joule heating. These intensive properties are related to device efficiency through the material “figure-of-merit”, *zT* *=* *S*^2^*σT/κ*, where *T* is operating temperature. Near room temperature, bismuth telluride compounds with optimized carrier concentration have the best *zT* (Supplementary Note 1)^29^. Accordingly, powders of *n-*type Bi_2_Te_2.73_Se_0.3_ and *p-*type Pb doped Bi_0.5_Sb_1.5_Te_3_, with respective optimal alloy compositions, were used in thread production. For comparison, the *zT* of inductively hot-pressed pellets of the same powders (Supplementary note 2) with carrier concentration levels of order ~10^19^ cm^−3^ were *zT* = 0.5 and 1.1 for *n*- and *p*-type, respectively.

**Fig. 1:**
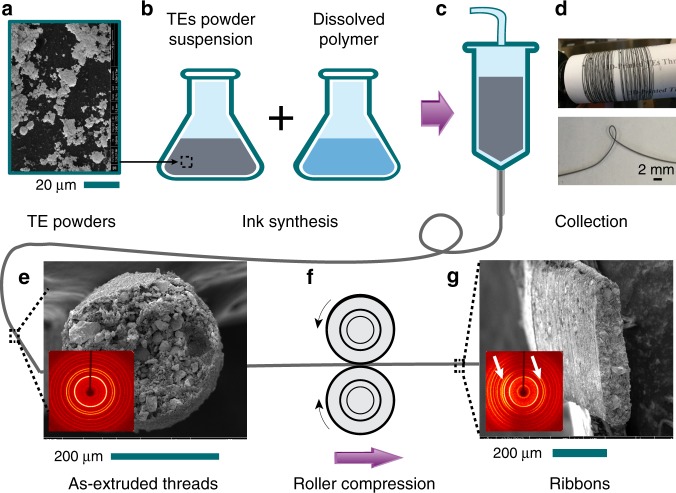
Schematic diagrams of continuous thermoelectric threads production. **a** Both *p*- and *n*-type Bi_2_Te_3_ powders, size <20 µm, are dispersed in the **b** polymer solution to form composite paints, respectively. **c** 3D-paints with proper viscosity are extruded out through a needle at room temperature, and **d** continuous threads with a diameter of 250 ± 40 µm are collected on a cardboard roller with a collection speed of 4 cm/s. The bending radius of as-extruded threads was less than 2 mm. **e** The cross-section surface morphology of extruded circular threads with fill volume fraction *Φ*(0) = 54 vol% reveals an X-ray diffraction pattern (inset) for random dispersed powders described by the Herman’s orientation parameter *f* *=* 0%; in comparison, **f** through the paired rollers, **g** the compressed ribbon with volume fraction *Φ*(*p*_max_) = 65 vol% reveals a diffraction pattern (inset) indicating oriented powders along thread direction with *f* *=* 30%. The white arrows point out the arcs used for orientation measurements.

Flexible thermoelectric threads were fabricated using a 3D-extrusion technique, as shown in Fig. 1. The powders (Fig. 1a) were dispersed in dichloromethane, and mixed with a small amount of dissolved nonconductive polylactide-co-glycolide to synthesize composite 3D-paints (Fig. 1b). The mixture was prepared immediately prior to extrusion to guarantee homogeneity, and volume ratios of micrograins to polymer binder of 80:20, 85:15, and 90:10 were tested for both *p*-type and *n*-type threads, with the highest performance achieved in 90:10 for *p*-type and 80:20 for *n*-type threads, respectively. These were subsequently used for thread extrusion through a 3D-printer (Fig. 1c). The continuous threads were collected on a cardboard spool (Fig. 1d) for further characterization. These threads showed good flexibility with a bending radius less than *r*_*b*_ = 2 mm, demonstrating the composite 3D-paint formulation and extrusion strategy as a feasible solution to produce continuous and flexible TE threads.

The compression testing of as-extruded threads determined the elastic modulus of *p*-type and *n*-type threads to be *E* = 23 ± 1 kPa and 11 ± 0.8 kPa, respectively. The higher modulus of *p*-type threads results from the additional 10 vol% of bismuth telluride powder (Supplementary Note 3).

### Compressed TE threads for enhanced performance

The conductivity is what limits of energy-conversion for such composite TE threads. It is important that the binder that provides elasticity is insulating, so that the conducting pathways are limited to that of the connected thermoelectric micrograins, rather than short-circuiting these micrograins^30^, but it is, of course, also critical that the grains themselves form well-connected pathways for conduction. Not surprisingly, the initial conductivity of as-extruded TE threads is very low, sometimes below the stable measurement threshold of *σ*_min_ = 10^−9^ S/cm, because of the significant porosity around *π* = 41 ± 5 vol% according to volumetric analysis, as-extruded after solvent evaporation. This porous state leads to sparse contacts among micrograins, as shown in the cylindrical cross-section of diameter *d* = 250 µm in Fig. 1e and side view in Supplementary Note 4. The diameter varies only by ± 40 µm in a 1-m length. Lateral compression through paired compression rollers can reduce this void volume fraction for mass production (Fig. 1f).

An in situ vise (Supplementary Note 5) was designed and assembled to enable the measurement of Seebeck coefficient and electrical conductivity under controlled lateral pressure via manual adjustment of spring compression, resulting in a ribbon of width *w* = 500 ± 40 μm and thickness *t* = 100 ± 10 μm. Conductivity was measured with standard lock-in amplifier techniques at *f* = 7 Hz shown in Supplementary Note 6, with a four-point method to eliminate contact resistance effects between the threads and the electrodes. The Seebeck coefficient was measured using the top and the bottom flat electrodes placed in contact with two heating blocks to produce a temperature gradient parallel to the wire^31,32^.

**Fig. 2:**
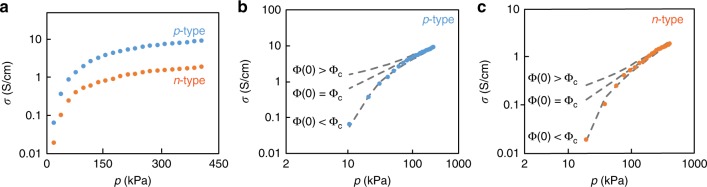
The electrical conductivity of composite threads was improved under compression. **a** The electrical conductivity *σ* of both *n*-type and *p*-type threads were improved with increasing applied compression pressures *p* in a log-linear plot. **b**, **c** Log-log plots demonstrate an asymptotic power-law relation between conductivity and pressure at high pressures. For the pure theoretical power-law fit line (central gray dash) the volume fraction is equal to the critical value with power-law exponent *s* = 0.87 (0.90) for *p*- (*n*-) type threads according to modified percolation theory in Eq. 2. The downward trend of experimental data matches the fit lower gray dash curve, implying that the initial concentration is just below the percolation threshold for conduction *Φ*(0) < *Φ*_c_. Conversely, if the conductivity were to saturate at low pressure (upper gray dash curves), this would imply that the initial concentration exceeded the percolation threshold *Φ*(0) > *Φ*_c_.

The conductivity was observed to greatly increase under applied lateral pressure as shown in Fig. 2a. Characterization was started at an initial pressure of *p* = 20 kPa to obtain a stable measurement. The conductivity was enhanced by a remarkable seven orders of magnitude to a maximum value of *σ* = 10 S/cm at pressure *p* = 400 kPa and temperature *T* = 80 ˚C. Figure 1e and g illustrate the change in cross-sectional morphology due to compression, deforming circular threads into pressed ribbons. According to volumetric analysis, the porosity *π* of pressed threads was observed to decrease to *π* = 18 ± 3 vol% as measured from changes in the cross-sectional area.

Theoretically, we turn to the percolation theory to quantify how the lateral compression increases conductivity. Intuitively, close contact between TE micrograins should be increased under compression, suggesting more available conducting pathways. Percolation theory predicts the following relation between conductivity *σ* and volume fraction of micrograins *Φ*

1 $$\sigma = \sigma _0\left[ {{\it{\Phi }}-{\it{\Phi }}_c} \right]^s$$

where *σ*_0_ denotes an electrical conductivity scale and *Φ*_c_ represents the percolation threshold. The model can be modified to predict a pressure *p* dependent conductivity *σ*(*p*) by introducing *Φ*(*p*) = *Φ*(0)/(1−*p*/*K*) as a pressure *p* dependent volume fraction of micrograins where *K* is the bulk modulus of the porous composite thread^33^. The resulting expression for pressure-dependent conductivity becomes

2 $$\sigma \left( p \right) = \sigma _0\left[ {\frac{{{\it{\Phi }} \left( 0 \right)}}{{1 - p/K}} - {\it{\Phi }} _{\mathrm{c}}} \right]^s$$

In the moderate pressure limit $\left[ {{\it{\Phi }}\left( 0 \right) - {\it{\Phi }}_{\mathrm{c}}} \right] < \frac{p}{K} < 1$, this expression asymptotically approaches the power-law $\sigma \left( p \right) \approx \sigma _0\left[ {{\it{\Phi }} \left( 0 \right)p/K} \right]^s$^33^. Accordingly, the data is plotted in a log-log plot and at high pressures is observed to asymptotically approach such power law (central gray line in Fig. 2b, c). Note that when the zero pressure volume fraction exceeds the critical value *Φ*(0) > *Φ*_c_, the conductivity saturates to a constant value at low pressure (upper gray curve), whereas when the volume fraction is less than the critical value *Φ*(0) < *Φ*_c_, there is a threshold pressure below which the conductivity is zero (lower gray curve in Fig. 2b, c). The downward trend of our experimental data is consistent with the latter case, such that the volume fraction of micrograin *Φ*(0) = 48 and 54 vol % that fit those curves are less than the observed percolation threshold *Φ*_c_, 51 and 56 vol % for *n*- and *p*-type, respectively. The remaining parameters of the fit are σ_0_ = 1350 and 820 S/cm for *n*- and *p*-type, respectively, comparable to the values *σ*_0 _= 1500 and 946 S/cm for annealed bulk pellets of the same material. The exponents *s* = 0.9 (0.87) for *n*- (*p*-) type threads depend on the material properties of TE micrograins, such as conductivity and size distribution, indicating similarity for both dopant types^30^. The fit of materials modulus *K* = 1.7 × 10^6^ and 1.6 × 10^6^ Pa for *n*- and *p*-type threads suggest the moduli are much higher than the testing pressure range. The compression decreases the porosity of threads *π* *=* 1−*Φ*(*p*)/c where the polymer-to-binder volume ratio *c* is 0.9 and 0.8 for *n*- and *p*-type, respectively. The fitted porosity π is 0.20 (0.18) for *n*- (*p*-) type threads under pressure *p* = 400 kPa at room temperature, which is comparable to the measured porosities 18 vol%. For the sake of the present work we focus on the maximum achievable electrical conductivity of pressed *n-* and *p-*type powders under pressure *p* = 400 kPa, which is *σ* = 6 ± 0.8 and 1.7 ± 0.6 S/cm, respectively.

Because bismuth telluride micrograins tend to be anisotropic in shape, it is worthwhile to quantify the effect of compression on grain alignment. Bismuth telluride micrograins are anisotropic with higher carrier mobility within their ab basal plane in comparison with the perpendicular *c* direction^34^. Owing to the easy cleavage of these materials along their *ab* planes, grains are typically plate-like with their shortest dimension parallel to the *c*-axis. According to the x-ray diffraction (XRD) patterns in Fig. 1e, the as-extruded thread with circular cross-section presented no preferential orientation as evidenced by the uniform rings. In contrast, the diffraction pattern from the perpendicular cross-section of a pressed ribbon (Fig. 1g) has bright arches at 2*θ* = 34.6˚ and 38.0˚ corresponding to (1,0,10) and (1,1,0) crystal planes, respectively, indicating that the grains have their *c*-axis preferentially oriented normal to the long axis of the ribbon. This implies that the higher conductivity *ab* planes are oriented along the ribbon. The alignment was evaluated by the degree of orientation *Π* *=* 54% and Herman’s orientation parameter *f* *=* 30%^35,36^, respectively, based on Fig. 1g. The calculations are described in the SI. Thus, it is apparent that compression also improves the conductivity by preferentially orienting the TE micrograins along the conduction direction. Quantitatively, this reorientation may only be a small contribution to conductivity relative to the percolation contribution since according to the literature the optimal improvement could account for at most a factor of 2 (*p-*type) to 4(*n-*type) for completely oriented *ab* plane grains^34^.

### Transport properties of composite TE threads

**Fig. 3:**
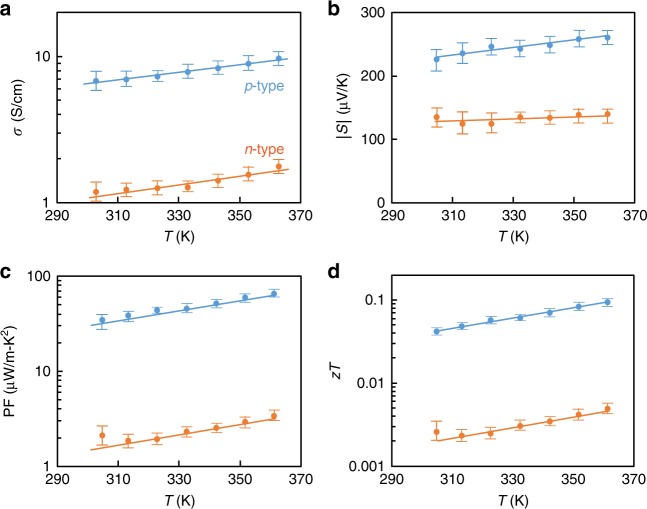
The transport properties of thermoelectric threads. Under pressure *p* = 400 kPa, **a** the electrical conductivity, **b** absolute Seebeck coefficient, **c** power factor, and **d** material *zT* with standard deviation errors, varied with increasing temperatures *T*, ranging from 300 K to 360 K. The measured thermal conductivity at room temperature of both *p*- and *n*-type threads was *κ* = 0.25 W/mK which is favorably lower than that of bulk compressed TE pellets, *κ* = 1.10 W/mK. Legend: blue is *p-*type and orange is *n-*type.

The transport properties of single threads were characterized from room temperature to 360 K to keep below the polymer binder degradation temperature while evaluating the thermoelectric performance, as shown in Fig. 3. The conductivity and Seebeck coefficient data were collected under a constantly applied pressure of *p* = 400 kPa. The conductivity was found to increase with temperature over the range of measurement; contrary to the standard hot-pressed power samples of the same micrograins where conductivity decreased with temperature^37,38^. Polymers typically demonstrate a reduced elastic modulus *K* with increasing temperature, so this increase in conductivity may result from the same pressure generating greater volume fraction *Φ* according to the percolation model, above. The conductivity of threads after cooling to room temperature and relieving external pressure decreased by only 10~15%. This partial recovery presumably indicates the residual elastic deformation of the composite under zero external pressure.

In addition to conductivity, the Seebeck coefficient *S* was also measured in order to quantify the power factor *σS*^*2*^. The Seebeck coefficients do not vary with pressure since they are independent of the extent of particle contact. The Seebeck coefficients of the composite threads in Fig. 3b are observed to be comparable to those of the hot-pressed TE powders, *p-*type *S* = 240 µV/K and *n*-type *S* = −120 µV/K^39^. The polymer is insulating, so it does not have any meaningful contribution to the Seebeck coefficient. The Seebeck of the TE micrograins depends on temperature and charge carrier doping, increasing with temperature as observed in Fig. 3b. Since the Seebeck coefficient of the composite is principally set by the Seebeck coefficient of the TE fillers themselves, any increase in the power factor and figure of merit must result from improvements in the conductivity, Fig. 3c, d. The final term in the figure of merit is the thermal conductivity *κ* = 0.25 W/mK, which was measured in a bulk composite pellet with identical composition to the threads. This value is 4.4 times lower than that of pure sintered Bi_2_Te_3_ from the same batch, *κ* = 1.1 W/mK. The reduction in value comes partly from polymer binders with a relatively low thermal conductivity (*κ* = 0.5 W/mK)^40^, and partly from the porosity. Accordingly, the calculated figure of merit of the compressed *p-*type thread is, *zT* = 0.08 at *T* = 360 K which is still lower than that of bulk pellets *zT* = 1.11 at the same temperature. Similarly, the figure of merit of the *n-*type thread is lower *zT* = 0.005 compared to that of bulk pellets *zT* *=* 0.54 at *T* = 360 K. Though it is expected that further compression will lead to improvement of the electrical conductivity and bring *zT*s even closer to the bulk values, the functionality here is sufficient to demonstrate proof of concept of a double-leg flexible thread thermocouple device, below.

Our work on polymer composite thermoelectric threads stands out in comparison with competing technology for flexible thermoelectric platforms. In Table 1, we show that alternative flexible thermoelectrics fall short since they are either fabricated with a technology that cannot be potentially scaled to industrial levels, or they demonstrate only limited *p-* or *n-*type function. Note also that some reported *zT* values are misleadingly high, since they are reported as bulk values but are in fact measured as thin films that require a supporting substrate. Such films are too fragile to survive without the substrate so real devices cannot be fabricated with these reported *zT* values, since the structural support will serve as a thermal short circuit.

**Table 1:** Comparisons between various techniques to produce flexible thermoelctrics.

Geometry	Thickness	Material	Process	*p-*type *zT*	*n-*type z*T*	Scalable	Free-standing
Thin film^9^	<10 µm	CuI	Vapor-deposition	0.20*	N/A	Y	N
Thin film^8^	<10 µm	HgTe, HgSe	Spin-coating	0.01*	0.90*	N	N
Thin film^16,17^	<10 µm	Conducting polymers: PEDOT:PSS	Organic synthesis	0.25*	N/A	Y	N
Thin film^21^	<10 µm	Conducting polymer composite: PEDOT:PSS/ Bi_2_Te_3_	Electrodeposition	0.02*	N/A	Y	N
Thin film^22^	<10 µm	Conducting polymer composite: PEDOT:PSS/ Bi_2_Te_3_	Spin-coating	0.20*	N/A	Y	N
Twisted yarns^14^	~ 1 mm	Polymer fiber coated with Bi_2_Te_3_	Vacuum sputtering	0.24	0.07	N	N
Film^19^	>100 µm	Nonconductive polymer composite: PVDF/Bi_2_Te_3_	Screen-printing	N/A	0.02	Y	Y
Threads (this work)	0.1~ 1 mm	Nonconductive polymer/Bi_2_Te_3_	3D-printing	0.08	0.005	Y	Y

*Note: Most film samples require a substrate for structural support, but these references calculate *zT* as though the substrate were not present

The figure of merit, processing scalability and free-standing structure of 3D-extruded continuous TE threads from this work (bottom row) were compared with that of alternate techniques. “Scalable” refers to the possibility of mass production at an industrial scale for functional devices. “Free-standing” means that underlying structural support is not necessary in order to make a useful device

### Flexibility and reliability

**Fig. 4:**
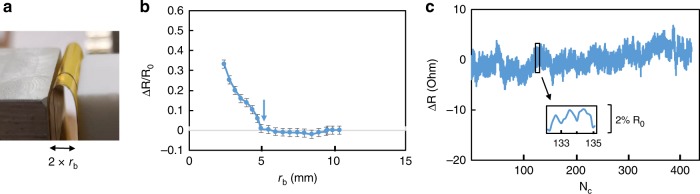
Bending studies of the thermoelectric threads. **a** The picture indicates a bending radius *r*_b_ = 5 mm of *p*-type thread held over a flexible Kapton plastic support. **b** The normalized electrical resistance change of a representative *p*-type thread was zero for bending radii larger than *r*_b_ = 5 mm and increased by 30% upon reducing to *r*_b_ = 2 mm. **c** The resistance shows no long term degradation even after hundreds of flexing cycles, indexed by *N*_c_.

The bending flexibility of representative *p*-type threads was characterized by the bending radius (*r*_b_) of curvature and the reliability was characterized by indexing hundreds of bends (*N*_c_), as shown in Fig. 4. The threads were mounted between Kapton films serving as a guide for the bending radius. The electrical resistance change (*ΔR*) with a bending deformation was normalized relative to the initial resistance (*R*_0_), is a function of bending radius. The resistance was roughly unchanged at the initial bending, and beyond the critical bending radius of *r*_b_ = 5 mm, resistance started to increase. The threads were on a neutral plane, and therefore there was no bending strain on the surface^41^. Regarding free-standing threads without supporting substrates, the critical bending radius was *r*_b_ = 44 mm in Supplementary Note 7.

Reliability testing indicated the resistance of *p*-type threads increased by less than 2% after a sequence of hundreds of bends with a bending radius, *r*_b_ = 6 mm or greater. The Seebeck coefficient of the *p*-type threads was *S* = 225 ± 12 µV/K remeasured after 400 reciprocal bending cycles at *r*_b_ = 6 mm and no obvious changes were observed in comparison to initial unbent threads.

### Flexible TE module

**Fig. 5:**
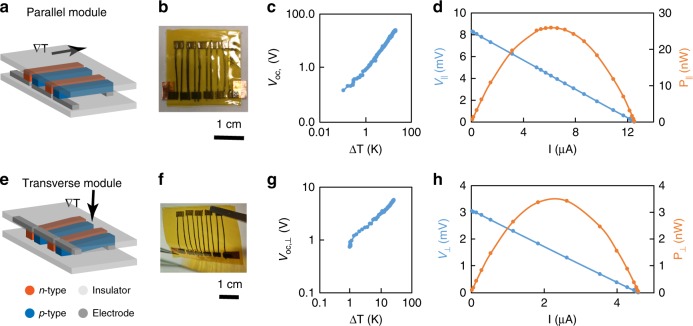
The thermoelectric performance of parallel and transverse TE modules. **a**, **b**, **e**, **f** schematics indicate two module configurations with thermal gradient parallel and transverse to the thread direction, respectively. Actual TE flexible modules contain five alternating parallel couples in series. The length of a single thread is *l* *=* 15 mm, and the thickness is *t* = 50 ± 10 µm which is the effective leg length for the thermal gradient in the transverse module. (**c**) and (**g**) are the open-circuit voltage (*V*_oc_) of parallel and transverse modules which monotonically increase with the temperature difference (notated with thermal gradient vector ∇T). (**d**) and (**h**) indicate the power output *P* is a function of the current and voltage, and the optimal was obtained at matched external resistance with the material resistance of the modules. The peak power for parallel and transverse modules is *P*_||_ = 26.2 nW, *P*_⊥_ = 3.4 nW, and the power coefficient for the respective thermocouples is *P*_TC,||_ = 0.050 nW/K^2^ and *P*_TC_,_⊥_ = 0.007 nW/K^2^.

Flexible thermoelectric modules were manually fabricated to illustrate potential applications of TE threads for thermal management along parallel and transverse heat gradients, respectively (Fig. 5). Pressed *p*-type and *n*-type threads were alternatingly patterned on Kapton substrates, and carbon paper with a thickness *t* = 100 µm was used as a flexible electrode material. Multiple modules were successfully fabricated, and each module consists of multiple thermocouples, demonstrating reproducibility at a device-level. The contact resistance between the threads and the carbon paper electrode was less than 1% of the resistance of the threads.

Power optimization can be achieved by balancing the current and voltage with proper external loads (Supplementary Note 8). A flexible module composed of 5 thermocouples connected in series was fabricated for testing with a temperature difference *ΔT* *=* 10 K. Two configurations were tested—one with the temperature difference parallel to the wire direction (||, Fig. 5a, b), and the other with the temperature difference transverse to the wire direction (⊥, Fig. 5e, f). Whereas the former device structure gives a higher energy conversion (Fig. 5c, d), the latter is more representative of a fabric that can be wrapped around a heat source (Figure g, h). The open-circuit voltage *V* and short-circuit current *I* were scanned to find the maximum power (Fig. 5d, h), as a function of external load *R*. The optimized power, *P*_||  _= 26.2 nW, *P*_⊥  _= 3.4 nW for *N* = 5 thread thermocouples was determined by tuning the resistive load, yielding a power coefficient for each thermocouple of *P*_TC,||_ = 0.050 nW/K^2^ and *P*_TC,⊥_ = 0.007 nW/K^2^, where the power can be expressed as, *P* = *P*_TC_ Δ*T*^2^*N*.

The fabrication of continuous threads by 3D extrusion followed by paired roller compression realizes a scalable production strategy, demonstrated in Supplementary Note 9. Toward high-performance flexible composite thermoelectrics, more work needs to be done to improve the electrical conductivity, which is still lower than that of the bulk counterparts. Binder material selection is critical, for example, a thermally-stable polymer binder allows annealing of semiconductors. We note, as well, that tensile strength was too low to be reliably measured. This is likely due to the porous structure and the relatively low modulus of polylactide-co-glycolide as a binder. Studies of new composites beyond the scope of this work will be required to increase tensile strength.

## Discussion

In conclusion, continuous flexible *n-* and *p-*type thermoelectric threads were successfully fabricated using a 3D-printing technique. Flexibility was achieved by dispersing TE micrograins within a minority amount of nonconductive polymeric matrix to create TE composites. The compression was demonstrated to be an essential method of improving the electrical conductivity of the composite threads by increasing intimate contact and preferential alignment of TE micrograins. Because of plastic deformation, the compression permanently alters the conductivity and therefore serves as a critical step to enhancing the performance of these thermoelectric threads. These threads could help extend potential applications of TE devices for wearable energy harvesting and ambient-powered internet-of-things near room temperature. A scalable, low-cost approach to producing a flexible thermoelectric module with alternating threads was also developed and demonstrated the potential of these TE threads for device applications.

## Methods

The thermoelectric powders were produced targeting a maximum *zT* with an optimized carrier concentration. Bismuth (99.999+%, Alfa Aesar), antimony (99.999%, Alfa Aesar), tellurium (99.999%, Alfa Aesar), selenium (99.999%, Alfa Aesar), and lead (99.9999%, Alfa Aesar) were used to synthesize a *p*-type compound with a nominal composition of Pb_*x*_(Bi_0.5_Sb_1.5_)_1−*x*_Te_3_ with *x* = 0.0025, and *n*-type with a composition of Bi_2_Te_2.73_Se_0.3_. These elements in glass tubes were sealed under a vacuum pressure less than *P* = 5 × 10^−5^ Torr after being solution cleaned and flame dried. Elements were heated at a rate of 100 °C/hr to melt and react, and they were held for 12 h at 800 °C before water quenching. The quenched ingot was powdered using a high energy ball milling, and the powders were then sieved to reject any particles larger than 20 µm. Nonconductive polymer, polylactide-co-glycolide, and micrograins as well were dispersed in excess dichloromethane with a smaller relative volume of 2-butoxy ethanol, a common surfactant, and dibutyl phthalate, an effective plasticizer. Composite 3D paints were extruded forming thermoelectric threads through a 3D BioPlotter (Envision TEC, MI, US) with a motor collecting at a rate, *v* = 8.4 cm/s, at ambient temperature. To characterize thermoelectric performance under various pressure, the in situ vice was machined with Macor, and a copper foil was used as electrodes. Compression mechanical properties of the threads were characterized by a dynamic mechanical analyzer (RSA-G2, TA, USA). Tensile strength was low because the threads were easily broken upon clamping into the test machine. The thermal diffusivity *a* = 0.42 mm^2^/s of both *n*-type and *p*-type composites was measured on a disc-shaped sample *r* = 1.2 cm on LFA (MicroFlash 457, Netzsch, Germany). The density was *d* = 3.9 g cm^−3^ and 3.8 g cm^−3^ for *n*-type and *p*-type materials, respectively. The porosity of composite threads was measured by the volumetric method, where the respective masses are compared to the known material densities, which in turn are compared to the overall thread volume. The specific heat capacity *C* = 0.16 J/(gK) was calculated following the law of Dulong and Petit^42^. The thermal conductivity *κ* *=* *adC* was therefore calculated. The TE module was fabricated with extruded threads that were compressed by lateral pressure. Paired compression-rollers realize a scalable production strategy of pressed threads.

## Supplementary information

Supplementary Information

Description of Additional Supplementary Files

Supplementary Movie 1

## Data Availability

The authors declare that the main data supporting the findings of this study are available within the article and its Supplementary Information files.
