# Supplementary material for: 3D extruded composite thermoelectric threads for flexible energy harvesting
Source: Nat Commun. 2019 Dec 6;10:5590. doi: 10.1038/s41467-019-13461-2 (PMC6897922; doi:10.1038/s41467-019-13461-2)
Supplement: Supplementary file 1 — Supplementary Information [file 41467_2019_13461_MOESM1_ESM.pdf]

Supplementary Information

3D Extruded Composite Thermoelectric Threads for Flexible Energy Harvesting

*J. Peng [1,2,] *, I. Witting[2], N. Geisendorfer[2], M. Wang[2], M. Chang[2], A. Jakus[3], C. Kenel[2], X. Yan[1], R. Shah [3, 4,5,] *, G. J. Snyder [2], and M. Grayson [1,]**

[1] Department of Electrical and Computer Engineering, Northwestern University, Evanston, IL, 60208, USA.

[2]Department of Materials Science and Engineering, Northwestern University, Evanston, IL, 60208, USA.

[3]Dimension Inx, LLC, Chicago, IL, 60611, USA.

[4]Department of Bioengineering, University of Illinois at Chicago, Chicago, IL, 60607, USA.

[5]Department of Biomedical Engineering, Northwestern University, Evanston, IL, 60208, USA.

Keywords: continuous thermoelectric threads, 3D-printing, bismuth telluride, flexible module

* Corresponding Author: mgrayson@eecs.northwestern.edu, jun.peng@northwestern.edu, ramilleshah@dimensioninx.com.

Supplementary Note 1. Material selections

A temperature difference can drive charge carrier transport in a variety of semiconductors, generating thermoelectric power. Thermoelectric materials have preferred working temperatures to achieve a high figure of merit. Thus, material selection is the first step to study material systems for a special application. For example, with wearable and portable electronics, the operating conditions are at or near room temperature. Bismuth telluride is, therefore, the best material selection to perform at ambient temperature, $T = 300$ K.

Doping levels and carrier concentrations also impact material properties and therefore the conversion efficiency and performance. An effective mass model was used to predict the material figure of merit zT and power factor (PF) with different levels of carrier concentrations. Supplementary Figure 1 demonstrates the well-known compromise between a large Seebeck coefficient and a high electrical conductivity calculated for *p*-type bismuth telluride leading to a maximum zT with an optimized carrier concentration, 1.6×10^{19} cm^{-3}.

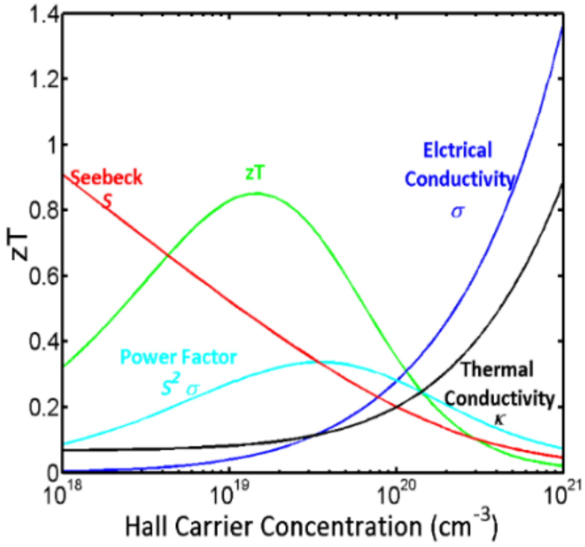

Supplementary Figure 1. Optimizing the efficiency (zT) based on the effective mass model. [1] The *p*-type thermoelectric bulk pellets of $Bi_{0.5}Sb_{1.5}Te_3$ obtains the thermal conductivity (κ; plotted on the y-axis from 0 to a top value of 13 Wm^{-1} K^{-1}), the Seebeck coefficient ($S = 0$ to 600 μVK^{-1}) and the electrical conductivity ($\sigma = 751$ to 1.7×10^7 S cm^{-1}).

Supplementary Note 2. Fabrication of *n*- and *p*-type powders

Bulk *n*-type $Bi_2Te_{2.73}Se_{0.3}$ and *p*-type Pb doped $Pb_x(Bi_{0.5}Sb_{1.5})_{1-x}Te_3$ with $x = 0.0025$ were synthesized using the Bridgman method, and followed by high energetic ball milling to produce micrograins with diameters less than 20 µm.

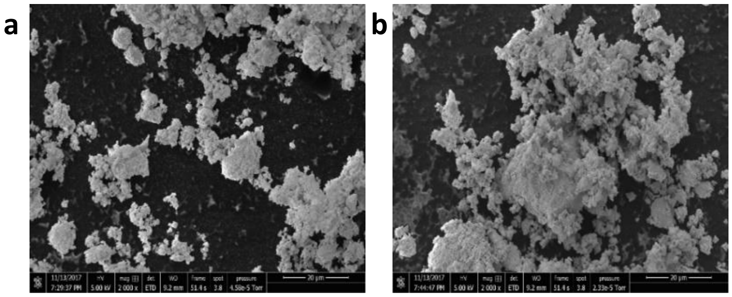

Supplementary Figure 2. The thermoelectric powder fillers. (a) *p*-type $Pb_x(Bi_{0.5}Sb_{1.5})_{1-x}Te_3$ and (b) *n*-type $Bi_2Te_{2.7}Se_{0.3}$ were prepared by a melt synthesis followed by a high energy ball milling. The scale bars are 20 µm, and the diameter of grains or clusters $d = 0.1{\sim}20$ µm.

Supplementary Note 3. Compression testing of composite threads

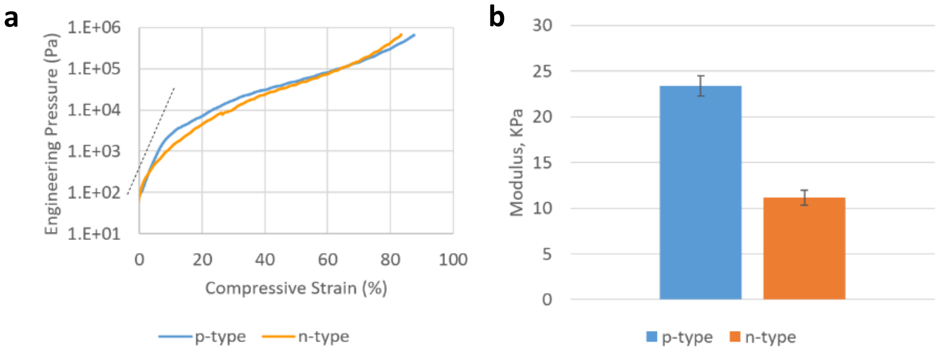

Supplementary Figure 3. The compression testing of as-extruded threads. (a) Engineering stress is a function of compressive strain and (b) Young's modulus of 23 ± 1 kPa and 11 ± 0.8 kPa for *p*-type and *n*-type threads, respectively.

Compression tests of the threads were performed at 80 °C to determine stress changes with strain. As-extruded circular threads with a diameter d = 200 µm were pressed to a ribbon with a thickness, t = 50~100 microns. Engineering stress was defined, force per unit area of the compression mold because the upper and bottom compression mold may contact during the testing.

The *p*-type threads obtain two times higher elastic modulus than that of *n*-type threads due to its higher volume ratio of thermoelectric powders. The elastic modulus was measured at the initial elastic range, strain less than 8.5 %. The volume ratios of micrograins to polymer binder were respectively 9:1 and 8:2 for *p*-type and *n*-type threads, which is the maximum ratios regarding extrusion processing.

The tensile properties of these threads are relatively low due to the relatively low modulus of polylactide-co-glycolide (~2 GPa) as a binder and the high volume ratio of micrograins. Both as-extruded circular threads and pressed ribbons were broken when clamping onto the tensile test machine. We are working on binder material selection to improve the tensile properties.

Supplementary Note 4. Extrusion of flexible thermoelectric threads

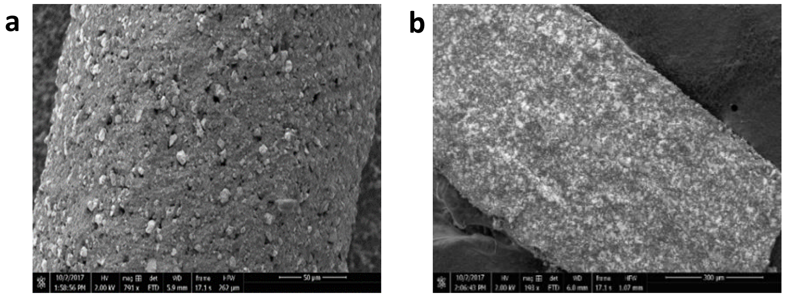

Supplementary Figure 4. Comparisons of the surfaces of as-extruded and pressed threads. When initially extruded, *p*-type circular threads have a rough fracture surface with loosely attached particles (a), while the compressed ribbons (b) present a denser surface with more contact between TE particles. The circular thread demonstrates a diameter d = 235 µm (area A = .043 mm^2) and the pressed ribbon shows a width w = 625 µm and a thickness t = 72 µm (A = .045 mm^2). Recall, the volume ratio is 9:1.

The printing ink was prepared using commercially available polymer precursor materials. The materials were mixed following solvent evaporation in room-temperature conditions. Thermoelectric (TE) powders were dispersed in dichloromethane (DCM) with ethylene glycol butyl ether (EGBE) and dibutyl phthalate (DBP) in concentrations η = 0.9 and 0.45 g per cubic centimeter of Bi_2Te_3, respectively. EGBE was used because it is a common surfactant and dibutyl phthalate is an effective plasticizer. The polylactide-co-glycolide (PLG), a polymer binder, was also dissolved in excess DCM. The PLG solution and powder dispersion were combined and thoroughly mixed until homogeneous. Excess DCM was evaporated until a quasi-static shear rate

viscosity of approximately 30 Pa·s was achieved. A higher powder concentration creates a better conductivity, which is critical to the TE efficiency. Inks with maximum weight ratios of 9:1 for *p*-type and 8:2 for *n*-type powders were prepared and sealed for printing. The composite threads were produced by 3D printing at room temperature. Continuous composite threads with a small bending radius (~2 mm) were extruded and collected on a cardboard roller.

Supplementary Note 5. Compression mold design

An *in-situ* vice was designed for soft or flexible samples sensitive to pressure and temperature.

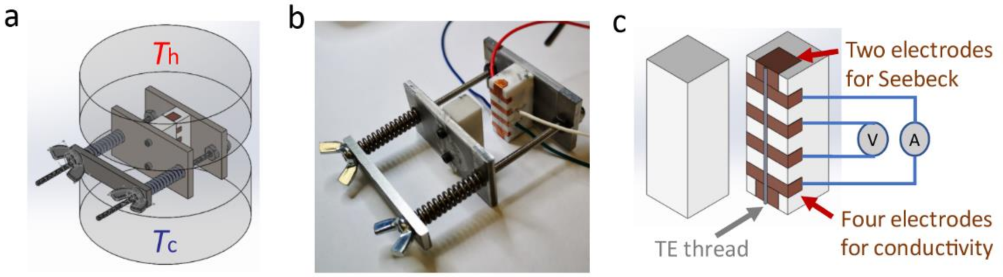

Supplementary Figure 5. Compression mold design for characterizations of pressure effects. (a) An *in-situ* vice. (b) The pressure was controlled by manual adjustment of the spring heights. (c) Multiple flat electrodes were embedded in compression mold for Seebeck and electrical conductivity measurement.

Supplementary Note 6. Measurements of electrical conductivity

A four-point method was applied to eliminate contact effects during electrical conductivity measurement. Lock-in amplifier 1 set the overall input voltage V_{ac} and lock-in amplifier measures the voltage over resistor R_{15}. The resistance $R_{AB} = V_A/10/(V_{CD}/R_{15})$.

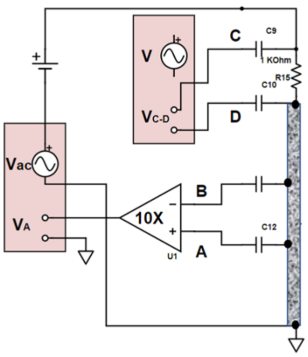

Supplementary Figure 6. AC circuit design for electrical conductivity measurement.

Supplementary Note 7. Particle Orientation Calculation Based on X-Ray Diffraction Pattern

X-ray diffraction patterns were obtained with a Bruker Diffractometer (Molly, Bruker, U.S.) by irradiating the composite thread with Mo Kα radiation perpendicular to the thread axis. Powder orientation was determined by identifying arcs with the highest level of equatorial diffraction, those corresponding to the (1,0,10) plane. Two approaches were employed to describe the powder orientation: the degree of orientation *Π* and Herman's orientation parameter *f* defined by Supplementary Equations 1, 2, and 3. [2]

$$\Pi = \frac{180° - \text{FWHM}}{180°} \quad (1)$$

$$f = \frac{3\langle \cos^2\varphi \rangle - 1}{2} \quad (2)$$

$$\langle \cos^2\varphi \rangle = \frac{\sum_0^{\pi/2} I(\varphi)\sin\varphi\cos^2\varphi}{\sum_0^{\pi/2} I(\varphi)\sin\varphi} \quad (3)$$

where ϕ is the azimuthal angle and $I(\phi)$ is the intensity along the Debye-Scherrer ring. The value of f ranges between -0.5 and 1 for orientations that are perpendicular to and parallel to the fiber direction, respectively. Random orientations would yield a value of 0. The degree of orientation *Π* formula estimates the orientation by the full width at half-maximum (FWHM) measurements of the peaks in a plot of intensity (brightness) versus azimuthal angle at the relevant value of 2θ.

Supplementary Note 8. Successfully achieved free-standing threads

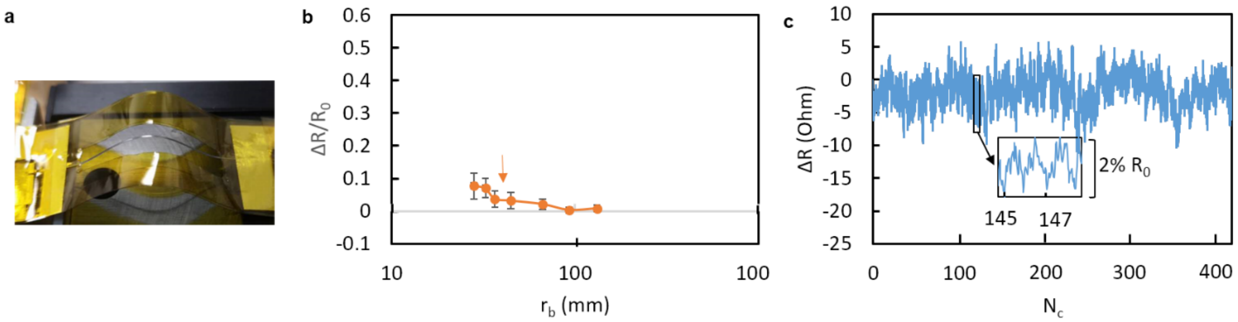

Supplementary Figure 7. The electrical conductivity and reliability of free-standing threads. (a) Bent *p*-type threads at r_b = 44 mm. (b)The normalized electrical resistance of free-standing *p*-type threads increases with a decreased bending radius. (c) the resistance is unaffected by hundreds of flexing cycles.

The compressed threads, with the ends attached on Kapton tapes were characterized by the electrical resistance change with respect to the bending radius, r_b. The electrical resistance change was defined as the ratio of the resistance change (ΔR) over the original resistance (R_0), $\Delta R / R_0$. A testing device was designed and assembled to characterized the bending radius and the bending reliability after hundreds of bends. The device was driven by a Servo motor and controlled by an Arduino controlled microcontroller.

Supplementary Note 9. Output current and voltage of two kinds of thermoelectric modules

Two configurations of flexible modules were manually patterned with 5 alternating thermocouples in serial, parallel module and perpendicular module with corresponding thermal gradients to the threads. The output currents and voltages with various external loads were scanned, indicating the existing of maximum output power. The optimized power output could be estimated $P = P_{TC}\,\Delta T^2 N$, based on the power coefficient for each thermocouple, $P_{TC,\parallel} = 0.050$ nW/K^2 and $P_{TC,\perp} = 0.007$ nW/K2, the thermocouple numbers (N), and the temperature difference (ΔT).

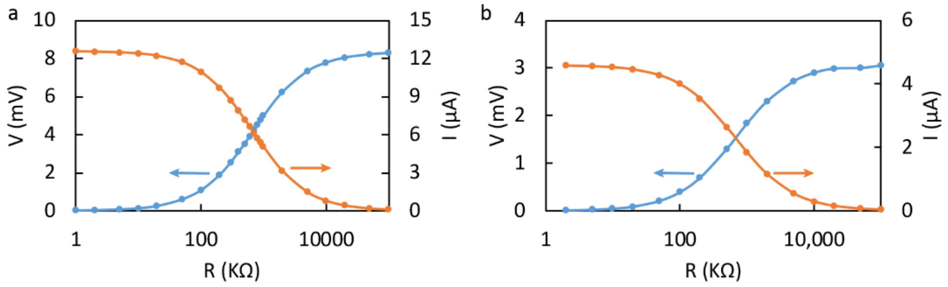

Supplementary Figure 8. Output performance of two kinds of thermoelectric modules. The output current I and voltage V were scanned with various external load resistors at a temperature difference $\Delta T = 10$ K: (a) parallel module and (b) perpendicular TE modules.

Supplementary Note 10. Scalability of the compression process

The scalability of the lateral compress process of continuous threads was demonstrated below in Supplementary Figure 9. The extruded thermal electric threads were pressed through a pair of compression rollers. The electrical conductivity properties of 3D extruded threads could be improved through compression by the paired rollers.

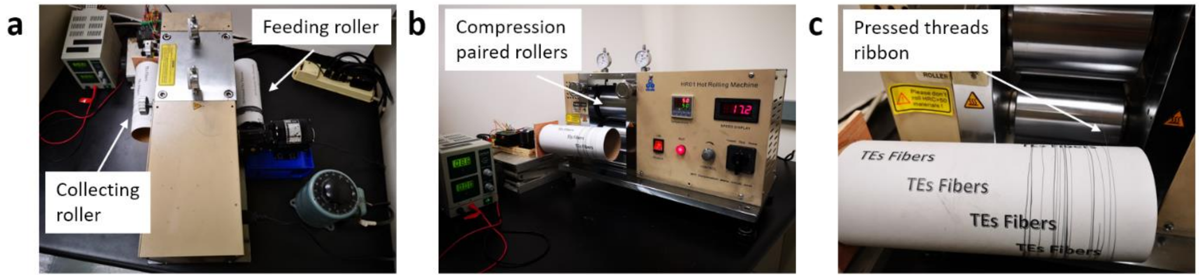

Supplementary Figure 9. Scalability of the compression process. A scalable compression process was demonstrated including a pressed-threads collecting roller driven by a speed-controlled step motor, and a feeding roller driven by a speed-controlled DC motor (a), and a compression roller (b). The pressed threads were wrapped up to a collecting roller (c). The paired compression rollers can control the pressing speed and the temperature, and the roller gap (thread thickness).

Supplementary References

1. Snyder, J., Toberer, E. Complex thermoelectric materials. *Nat. Mater.* **7**, 105-114 (2008).
2. Bacon, K. *Newer methods of polymer characterization*. Interscience Publishers (1964).
