# Supplementary material for: 3D extruded composite thermoelectric threads for flexible energy harvesting
Source: Nat Commun. 2019 Dec 6;10:5590. doi: 10.1038/s41467-019-13461-2 (PMC6897922; doi:10.1038/s41467-019-13461-2)
Supplement: Supplementary file 2 — Description of Additional Supplementary Files [file 41467_2019_13461_MOESM2_ESM.pdf]

Description of Additional Supplementary Files

File Name: Supplementary Movie
Description: Fabrications of Thermoelectric Threads.
